# Ventilation

**Published:** 1885-06

**Authors:** 


### Ventilation.

It is found that a man gives off somewhat over 6-10 of a cubic foot of carbonic acid per hour; that a lamp or two lighted candles produce the same amount, and that a gas jet, burning 3 cubic feet of gas per hour, produces as much carbonic acid per hour as two or three people. It is true that the gas gives off no organic impurities, but if not burning brightly the poisonous carbonic oxide is always formed. If we adopt 6 volumes in 10,000 as the safe limit of the amount of carbonic acid to air, then it follows that for every man or lamp or two candles in a room we must supply at least 1,000 cubic feet of pure air in every hour to dilute the 6 10 cubic foot of carbonic acid formed. A gas jet will require two or three times as much pure air. But since the admitted air contained carbonic acid we must supply more air to not exceed the maximum adopted; thus if the admitted air contain three volumes in 10,000 of carbonic acid, we must admit 2,000 cubic feet for every person, since the 6-10 of carbonic acid admitted, added to the 6-10 expired per hour, gives the ratio of 12 to 20,000 or 6 to 10,000 allowed. It is said by some that experience in hospitals shows that from 2,000 to 3,000 cubic feet of fresh air should be admitted every hour for each individual; whilst again we are told that for a healthy person in a barrack room 1,200 cubic feet per hour will suffice, and that the vitiation, tested by the sense of smell, for hospitals is not perceptible when somewhat less than the 2,000 to 3,000 cubic feet are provided. No fixed standard has thus been agreed upon. In fact, it doubtless varies with the climate and the health of the person. The Laplander can breathe impure air better than we, probably because the organic impurities thrown off by him are not so readily decomposed as in our warmer air. The carbonic acid formed by combustion and respiration being heavier than air at the same temperature, would sink to the floor ; but in consequence of its high temperature it first rises to the ceiling so that as much as 60 to 70 parts of it in 10,000 of air has been found at the top of an ordinary-sized room in which two people were sitting and three gas-jets burning. At the same temperature, however, we should expect to find the largest amount of it at low elevations, thus vitiating the lower strata of the atmosphere, or room, very greatly. Fortunately, however, gases have the power of "diffusion," so that a heavy gas will actually rise to mix with a lighter gas ; further, it will pass through membranes and thin plates of stucco to effect the same object, so that the amount of carbonic acid is not generally a function of the elevation of a locality. Where a room has no flue or chimney to keep up a constant circulation, then openings should be provided neor the top of the room to let the warmer impure gasses out, and not let them cool and descend again to vitiate the air we breathe.—*Gaillards Medical Journal.*

